# Correction to: Rapid Cycle Implementation and Retrospective Evaluation of a SARS-CoV-2 Checklist in Labor and Delivery

**DOI:** 10.1186/s12913-021-06868-5

**Published:** 2021-08-18

**Authors:** Liana Zucco, Nadav Levy, Yunping Li, Toni Golen, Scott A. Shainker, Philip E. Hess, Satya Krishna Ramachandran

**Affiliations:** 1 Department of Anesthesia, Critical Care and Pain Medicine, Beth Israel Deaconess Medical Center, 330 Brookline Avenue, Boston, MA 02215 USA; 2 Department of Obstetrics, Gynecology and Reproductive Medicine, Beth Israel Deaconess Medical Center, Boston, MA USA

**Correction to: BMC Health Serv Res 21, 775 (2021)**

**https://doi.org/10.1186/s12913-021-06787-5**

Following publication of the original article [1], the caption to Fig. 1 was inadvertently truncated. The correct caption should be:

Rapid cycles of change. Schematic representation of rapid-cycle implementation, demonstrating how the individual processes of inter-professional input, testing in real-time, focused debriefing, on-site walkthroughs and iterative re-design contributed to our final refined product. CFIR: Consolidated framework for implementation research, L&D: labor and delivery.

The original article [1] has been updated.
